# Comparative proteomics of cerebrospinal fluid reveals a predictive model for differential diagnosis of pneumococcal, meningococcal, and enteroviral meningitis, and novel putative therapeutic targets

**DOI:** 10.1186/1471-2164-16-S5-S11

**Published:** 2015-05-26

**Authors:** Ana Paula Cordeiro, Rosiane Aparecida Silva Pereira, Alex Chapeaurouge, Clarice Semião Coimbra, Jonas Perales, Guilherme Oliveira, Talitah Michel Sanchez Candiani, Roney Santos Coimbra

**Affiliations:** 1 Genomics and Computational Biology Group-Research Center René Rachou (CPqRR), Oswaldo Cruz Foundation (FIOCRUZ), 30190-002, Belo Horizonte, Brazil; 2 Laboratory of Toxinology, Oswaldo Cruz Institute, FIOCRUZ, 21040-360, Rio de Janeiro, Brazil; 3 Children's Hospital João Paulo II - FHEMIG, 30130-110, Belo Horizonte, Brazil; 4 Biosystems Informatics - Research Center René Rachou (CPqRR), Oswaldo Cruz Foundation (FIOCRUZ), 30190-002, Belo Horizonte, Brazil

**Keywords:** meningitis, differential diagnosis, therapeutic targets, comparative proteomics, pathway analysis, meningococcal meningitis, pneumococcal meningitis, enteroviral meningitis

## Abstract

**Background:**

Meningitis is the inflammation of the meninges in response to infection or chemical agents. While aseptic meningitis, most frequently caused by enteroviruses, is usually benign with a self-limiting course, bacterial meningitis remains associated with high morbidity and mortality rates, despite advances in antimicrobial therapy and intensive care. Fast and accurate differential diagnosis is crucial for assertive choice of the appropriate therapeutic approach for each form of meningitis.

**Methods:**

We used 2D-PAGE and mass spectrometry to identify the cerebrospinal fluid proteome specifically related to the host response to pneumococcal, meningococcal, and enteroviral meningitis. The disease-specific proteome signatures were inspected by pathway analysis.

**Results:**

Unique cerebrospinal fluid proteome signatures were found to the three aetiological forms of meningitis investigated, and a qualitative predictive model with four protein markers was developed for the differential diagnosis of these diseases. Nevertheless, pathway analysis of the disease-specific proteomes unveiled that Kallikrein-kinin system may play a crucial role in the pathophysiological mechanisms leading to brain damage in bacterial meningitis. Proteins taking part in this cellular process are proposed as putative targets to novel adjunctive therapies.

**Conclusions:**

Comparative proteomics of cerebrospinal fluid disclosed candidate biomarkers, which were combined in a qualitative and sequential predictive model with potential to improve the differential diagnosis of pneumococcal, meningococcal and enteroviral meningitis. Moreover, we present the first evidence of the possible implication of Kallikrein-kinin system in the pathophysiology of bacterial meningitis.

## Background

Meningitis is characterized by the inflammation of the leptomeningeal membranes that surround the brain and spinal cord [1-3]. Meningitis can be classified as aseptic or bacterial [1]. The term "aseptic meningitis" refers to meningeal inflammation without evidence of causative bacterial infection [4], which can be caused by viruses, fungi or chemical agents. Over 80% of all cases of viral meningitis are caused by enteroviruses, and present a benign clinical course being rarely associated with bad outcome [4,5]. As opposite, bacterial meningitis is characterized by an exacerbated inflammatory response in the central nervous system (CNS), and its main causative agents are *Streptococcus pneumoniae *e *Neisseria meningitidis *[1]. Bacterial meningitis is one of the 10 leading causes of death due to infectious diseases worldwide [6], and about 10% patients die within 24-48 h of the onset of symptoms. Long-term neurological sequelae occurs in 10-20% of survivors [6]. Brain damage in meningitis is due to a complex interplay between products released by the pathogens, such as pneumolysin, cell wall degradation products, and DNA, and the exacerbated host inflammatory response with the recruitment of neutrophils to the subarachnoid space. Activated neutrophils release many potentially cytotoxic agents including oxidants that can cause lipid peroxidation, DNA strand breakage, or matrix metalloproteinase (MMP) activation (reviewed in [7]).

Early clinical findings of meningitis are usually unspecific, which sometimes impairs the diagnosis. In addition, patients in the early stage of the disease or pre-treated with antibiotics may have normal or inconclusive cytochemical findings in the cerebrospinal fluid (CSF)[8-10]. Definitive diagnosis is usually established after CSF or blood culture, and/or detection of bacterial antigens by immunological tests, which may take longer than the therapeutic window. Thus, at present, the empiric treatment with broad-spectrum antibiotics, in some cases associated with the anti-inflammatory drug dexamethasone, is recommended to patients with suspected bacterial meningitis [11]. However, dexamethasone has been shown to aggravate neuronal apoptosis in the dentate gyrus of the hippocampus of animal models of bacterial meningitis [12,13]. Therefore, there is a need for novel robust and specific laboratory parameters to support early and accurate differential diagnosis of meningitis, a pre-requisite to the clinical decision for the appropriate therapeutic approach.

This study aimed at identifying CSF protein markers of the host response to the most prevalent forms of bacterial and enteroviral meningitis, as well as to disclose putative novel therapeutic targets to prevent mortality and morbidity associated with the malignant forms of this disease. Using 2-dimensional gel electrophoresis (2D-PAGE) and mass spectrometry (MS), four candidate biomarkers were identified and combined in a qualitative predictive model for the differential diagnosis of pneumococcal, meningococcal and enteroviral meningitis. Moreover, pathway analysis of the disease-specific proteome signatures unveiled pathways and biological processes involved the pathophysiological mechanisms leading to brain damage in bacterial meningitis. Putative novel therapeutic targets are proposed.

## Results

### 2D PAGE qualitative analysis and protein identification

Qualitative comparison of the CSF proteome of the groups with pneumococcal, meningococcal, and enteroviral meningitis to each other, and to the control group disclosed aetiology-specific spots characteristic of each form of meningitis (Additional Figures 1, 2, 3, 4, Additional files 1, 2, 3, 4; Additional Tables 1 - 3, Additional files 5, 6, 7).

Of the 695 protein spots subjected to mass spectrometry, 553 (80%) were identified, corresponding to 117 distinct proteins (Additional Table 4 Additional file 8). Because we targeted the host proteome, at least part of the non-identified spots in the 2D gels may be microbial proteins. Twenty identified proteins occurred in the intersection subsets of pneumococcal, meningococcal, or enteroviral meningitis. However, most of the aetiology-specific spots disclosed by comparing the 2D PAGE gels were ruled out as potential biomarkers after identification by mass spectrometry, since the same corresponding protein occurred in other positions of the gels (Additional Tables 1 - 3, Additional files 5, 6, 7). These changes in the migration properties of proteins at 2D PAGE gels may be explained by alternative splicing, or by posttranslational modifications. As a consequence, only the aetiology-specific spot of the intersection subset of patients with meningococcal meningitis corresponding to kininogen-1 was eligible to integrate the qualitative predictive model for differential diagnosis of meningitis. Six non aetiology-specific spots belonging to the intersection subsets of pneumococcal, and/or meningococcal, and/or enteroviral meningitis (corresponding to complement C3, c-reactive protein, and apolipoprotein A-I) proved to be informative to the predictive model.

### Predictive model for differential diagnosis of meningitis

**Figure 1:**
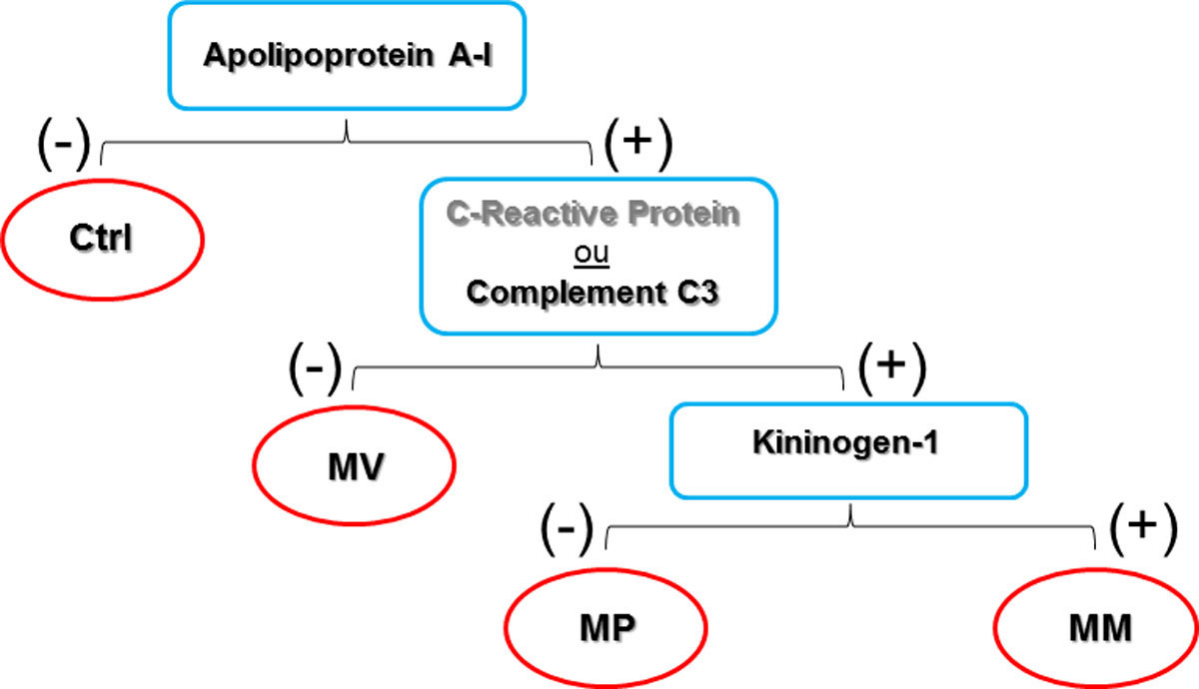
**Predictive model for the differential diagnosis of acute pneumococcal, meningococcal, and enteroviral meningitis**. PM = pneumococcal meningitis; MM = meningococcal meningitis; VM = enteroviral meningitis; CTRL = control.

A qualitative and sequential predictive model was proposed to differentiate pneumococcal from meningococcal meningitis and bacterial from enteroviral meningitis or controls. The qualitative predictive model consists of three nodes, each one related to the presence or absence in the CSF of at least one of four putative protein biomarkers. At the first node, the presence of Apolipoprotein A-I indicates patients with bacterial or enteroviral meningitis. At the second node, the presence of C-reactive protein, and complement C3 in the CSF are associated with the diagnosis of bacterial meningitis, and their absence points to enteroviral meningitis. Finally, at the third node, the presence of kininogen-1 is associated with meningococcal meningitis, and its absence, with pneumococcal meningitis (Table 1 and Figure 1).

**Table 1:** Group distribution of the proteins selected for composing the qualitative predictive model for differential diagnosis of meningitis.

Uniprot	Genbank	Definition	∩ PM	∩ MM	∩ VM	U PM	U MM	U VM	U Ctrl
P02647	90108664	Apolipoprotein A-I	1	1	1	1	1	1	0
P02741	1942435	C-reactive protein	1	1	0	1	1	0	1
P01024	179665	Complement C3	1	1	0	1	1	0	1
P01042	4504893	Kininogen-1	0	1	0	0	1	0	0

∩ = intersection subset; U = union set; MP = pneumococcal meningitis; MM = menigococcal meningitis; MV = enteroviral meningitis; Ctrl: control; 1 = present; 0 = absent.

### Subcellular localization of proteins

The subcellular localizations of the identified proteins were predicted using the web version of the SherLoc 2 software (http://abi.inf.uni-tuebingen.de/Services/SherLoc2). As expected, most proteins identified in the union sets of the groups of patients with meningitis were predicted to be located in the cytoplasm (46%) or at the extracellular space (45%). However, about 10% of these proteins were predicted to be located in the nucleus (3%), plasma membrane (4%) or mitochondria (2%). It is conceivable that these proteins were released into the CSF as a result of membrane rupture and cell death triggered by the inflammatory process. As opposite, the proteins belonging to the intersection subsets of groups of patients with meningitis were predicted be only extracellular (71%) or cytoplasmatic (29%), supporting the idea that they may play a role in the pathophysiological processes of host response to the infection of the CNS.

### Pathway analysis

The canonical pathway "Blood coagulation_Blood coagulation" was associated with meningococcal meningitis (*P *value: 6.38 × 10^-6^). The cellular process "Inflammation_IL-6 signalling" was associated with meningococal and enteroviral meningitis (*P *values< 10^-6^), but much more strongly associated with pneumococcal meningitis condition (*P *value: 1.07 × 10^-13^), while the "Inflammation_Kallikrein-kinin system", was associated only with meningococcal meningitis (*P *value: 1.7 × 10^-7^).

## Discussion

This is the first study to compare the CSF proteome profiles of patients with pneumococcal, meningococcal, or enteroviral meningitis and that of individuals without infection in the CNS or neurodegenerative or psychiatric diseases. We analysed individually the CSF samples of 24 subjects with pneumococcal, meningococcal, or enteroviral acute meningitis and controls without infection in the CNS or neurodegenerative or psychiatric diseases. This conservative strategy added confidence to our results since they represent the consensus of each group formed by six patients of both sexes, with different ages, and living in different areas of the northeast and southeast Brazil. Another level of confidence came from the proteins subcellular location prediction. The fact that all proteins belonging to the intersection subsets of the groups of patients with meningococcal, pneumococcal or enteroviral meningitis were predicted to be only extracellular (71%) or cytoplasmatic (29%) supports the idea that they may play a role in the pathophysiological processes of host response to the infection of the CNS.

We selected four proteins of the host inflammatory response to create a qualitative and sequential predictive model for differential diagnosis of meningococcal, pneumococcal and enteroviral acute meningitis. Evidences of the diagnostic potential of three of these biomarkers have already been published. For instance, the concentration of apolipoprotein A-I in the CSF of patients with meningitis increased in the acute phase of the disease, returning to basal concentration in the convalescent phase. In patients with other neurological diseases, the CSF levels of apolipoprotein A-I remained in the same range as in controls [14]. Our results also agree with other works reporting high concentrations of complement C3 in the CSF of patients with bacterial meningitis compared to patients with enteroviral meningitis and controls without infection in the CNS [15,16]. Besides, several studies have demonstrated the existence of increased levels of C-reactive protein in cerebrospinal fluid and serum of patients with bacterial meningitis compared with patients with viral meningitis and control subjects [17-20]. However, there is no indication in the literature of the use of apolipoprotein A-I, complement C3 and protein C-reactive for differentiation between patients with acute bacterial meningitis, patients with enteroviral meningitis, and individuals without infection in the CNS. In addition, our results point to kininogen-1 as a potential biomarker to differentiate between meningococcal and pneumococcal acute meningitis. This is the first report of increased CSF levels of kininogen-1 in patients with meningitis. The putative role of this protein in the host response to meningococcal meningitis is discussed below.

Our work is not the first attempt to investigate the CSF proteome of patients with meningitis. Jesse et al [21] had already performed comparative proteomic analysis using pools of CSF samples from groups of patients with viral or bacterial meningitis, the latter group included four different etiologic agents, namely: *N. meningitidis*, *S. pneumoniae*, *Staphylococcus aureus *and *Listeria monocytogenes*). These authors proposed six proteins as candidate markers for the differential diagnosis of bacterial and viral meningitis, three of them were also identified in the present study (haptoglobin, fibrinogen β chain and prostaglandin D synthase). However, in the present study, these proteins occurred sparsely in the union sets of the groups of patients with different forms of meningitis (data not shown), and therefore, were ineligible as candidate biomarkers for a qualitative model for differential diagnosis. In another work, Goonetilleke et al [22] compared the proteome of pools of CSF samples from control individuals with those from patients who died due to pneumococcal meningitis and survivors. These authors reported more than 2,400 proteins differentially expressed as a result of meningitis. Among the proteins found by Goonetilleke et al, 26% were also identified in our study, and a common marker of meningitis was selected, namely the complement C3. This protein had its expression level decreased in patients who died due to pneumococcal meningitis. The other proteins identified by Goonetilleke et al and also detected in the present study occurred sparsely in the union sets of patients with pneumococcal, meningococcal or viral meningitis and thus, were not considered as potential biomarkers.

Previous works have investigated the CSF levels of inflammatory cytokines and chemokines in patients with meningitis, such as IL-6, IL-1β, TNF-α, IL-10, chemokines of the CXC family (IL-8) and growth factors using immunoenzymatic methods [23-30]. None of these proteins were detected in the present study using 2D PAGE followed by mass spectrometry. This may be explained by the relative low sensitivity of the 2D PAGE method compared to immunologic methods used in the above mentioned works. However, it is indeed the relative low sensitivity of the 2D PAGE that ensures the robustness of our results, i.e. the qualitative differences detected in the proteomes of the investigated groups are probably due to marked differences in the CSF concentrations of these proteins as part of the pathogen-specific host responses. Consequently, the definition of concentration cut off points for the development of diagnostic kits using more sensitive platforms such as ELISA, latex agglutination, etc., should be facilitated by the magnitude of the differences in the concentrations of the selected biomarkers in the CSF of patients with pneumococcal, meningococcal, or enteroviral acute meningitis.

In order to shed light on the pathophysiological mechanisms underlying the distinct outcomes associated with the malign or benign forms of meningitis, we submitted the disease-specific proteome signatures to a pathway analysis using the software MetaCore from Thompson Reuters. Not surprisingly, blood coagulation was the canonical pathway most strongly associated with meningococcal meningitis. Indeed, activation of coagulation and attenuation of fibrinolysis in the CSF are important features of bacterial meningitis and may contribute to brain infarction [31]. Also, as expected, IL-6 signalling-mediated inflammation was the cellular process most strongly associated with pneumococcal meningitis. IL-6 contributes directly to blood brain barrier (BBB) permeability and boosts intracranial pressure and cerebral oedema in meningitis [32,33]. Nonetheless, pathway analysis unveiled an unexpected association between the kallikrein-kinin system (KKS) and meningococcal meningitis. Kininogen-1, the forth biomarker of the predictive model developed in this study is a constituent of the kallikrein-kinin system (KKS). Kininogen is a kinin precursor which binds to two receptor types, B1 and B2. While the B2 receptor is constitutively expressed in various cell types, including endothelial cells, the B1 receptor is inducible by proinflammatory agents, such as IL-1β, in pathophysiological conditions [34-36]. Both receptors are members of the G protein-coupled receptors subfamily [37] that, by binding to Gαi and Gαq families could regulate vascular processes, including the production of nitric oxide (NO) [35,36]. NO production via endothelial nitric oxide synthase (eNOS) allows B2-mediated regulation of blood pressure and maintains the normal endothelial barrier [38,39]. As opposite, in inflammatory conditions, the endothelial cells can express inducible nitric oxide synthase (iNOS) for NO production under cytokines stimulation, such as IL-6, IL-1β and IFN-γ. Although poorly understood, it is known that the NO production by iNOS can be also mediated by a B1 receptor in a process involving extracellular-regulated kinase (ERK) [40]. Martins et al [41] showed that the B2 kinin receptor has protective effects in the CNS, whereas the B1 receptor has toxic effects in this system. The stimulation of the B2 receptor has preserved the integrity of hippocampal pyramidal neurons against N-methyl-D-aspartate (NMDA) receptor-mediated excitotoxicity. While the B2 receptor blocking resulted in exacerbated brain damage in animal models of cerebral ischemia, the B1 receptor inactivation has promoted protection against myocardial and cerebral oedema in postischemic mice [42]. Thus, the protective role of KKS system appears to be preferentially associated with the B2 receptor, which regulates blood pressure, reduces the BBB permeability and decreases the release of pro-inflammatory cytokines [35,42,43]. In bacterial meningitis, the percentage of cases that progress to neurological sequelae ranges from 16 to 38% in pneumococcal meningitis and from 3 to 21% in meningococcal meningitis [44-46]. One possible explanation for the relatively lower incidence of neurological sequelae in meningococcal meningitis could be the activation of the KKS system that might provide a neuroprotective effect in the brain by reducing the vascular permeability and brain oedema.

## Conclusions

The host response to the invasion of the CNS by bacterial and viral pathogens leads to aetiology-specific proteome signatures in the CSF. Protein biomarkers with potential to improve the differential diagnosis as well as putative therapeutic targets for adjunctive therapy were predicted by comparing the CSF proteomes of groups of patients with acute meningitis caused by pneumococci, meningococci, or enteroviruses.

## Materials and methods

### Ethics statement

This project was conducted according to the principles expressed in the Declaration of Helsinki and was approved by the Brazilian Council for Research Ethics (registry numbers 25000.140699/2005-93 and 25000.199054/2008-18). All patients or their legal guardians received written information concerning the study and gave written informed consent for participation.

### Patients, and CSF samples pre-treatment

The cohort comprised six patients for each form of meningitis investigated, namely pneumococcal, meningococcal, and enteroviral meningitis, and six individuals without infection in the CNS, neurodegenerative, or psychiatric diseases. These 24 patients had been admitted at Hospital Giselda Trigueiro (Natal/RN) or Children's Hospital João Paulo II - FHEMIG (Belo Horizonte/MG). Cerebrospinal fluid (CSF) samples were collected by lumbar puncture before patients received any antibiotics or anti-inflammatory drug and were kept at 4°C. No more than six hours after puncture, CSF samples were centrifuged for 10 minutes at 5,000 rpm, and the supernatants were frozen at -20°C for up to one week and then at -80°C until further analysis.

Patients with bacterial meningitis had pneumococci, or meningococci in CSF, or blood specimens detected by culture, Gram-staining, and/or latex agglutination test. These patients showed CSF white blood cell counts greater than 100 cells/mm^3 ^being more than 50% neurophils. Patients with enteroviral meningitis had pleocytosis characterized mostly by mononuclear cells in the absence of detectable bacterial pathogen. Furthermore, RNA of enterovirus was detected in CSF by real time PCR as previously described [47]. One patient with viral meningitis had mild pleocytosis and no meningeal signs at the time CSF sample was collected, but tested positive for enterovirus at real time PCR. The control group was formed with individuals who underwent lumbar puncture because of suspected of meningitis, but have a normal CSF profile, spontaneous relief of symptoms. Additional details of the patients are shown in Table 2. CSF samples with more than 250 red blood cells/µL suggestive of puncture accident were excluded.

**Table 2:** Main characteristics of the groups of patients.

	PM	MM	VM	Ctrl
	
Region *				
Northeast	4	1	0	1
Southeast	2	5	6	5
Gender *				
Female	1	4	3	2
Male	5	2	3	4
**Age (years)**				
Median (range)	17.5 (2 - 36)	9.5 (<1 - 13)	6.0 (<1 - 8)	1.9 (<1 - 33)
**CSF parameters**				
WBC	5,600 (240 - 1,199)	4,680 (72 - 19,500)	27 (2 - 500)	2 (1 - 9)
% PMN	91 (80 - 95)	94 (5 - 96)	20 (0 - 73)	34 (0 - 94)
Protein (mg/dL) **	261 (110 - 474)	195 (66 - 461)	30 (18 - 38)	25 (20 - 35)
Glucose (mg/dL) **	11 (1 - 71)	22 (2 - 53)	50.5 (47 - 68)	55 (40 - 72)

PM = pneumococcal meningitis; MM = meningococcal meningitis; VM = enteroviral meningitis; Ctrl = control; WBC - White blood cell count; PMN = polymorphonuclear neutrophils; * = number of patients; ** median (range).

Proteins in aliquots of 300 µL of CSF were precipitated with acetone (Merck KGaA, Dramstadt, Germany), the pellets were ressuspended in 15µL ultrapure water and the volumes were adjusted to 100 µL with PBS buffer. These 3X concentrated protein solutions were applied to the columns provided with the Albumin & IgG Depletion SpinTrap kit (GE Healthcare, Uppsala, Sweden) to deplete overabundant proteins. The proteins in the whole volumes eluted from the columns (300 µL) were precipitated again with acetone and solubilized in 50 µL of IEF rehydration buffer [8 M Urea, 2 M Thiourea, 4% CHAPS, 0.0025%]. Protein concentrations were assessed using the Bradford assay [48]. Protein degradation was monitored in silver stained 1D PAGE gels before and after albumin and IgG depletion. None of the 24 samples showed signs of protein degradation.

### 2D-gel electrophoresis, imaging, and qualitative analysis

Two 2D-gels were run per each subject. Per each group, a total of 12 gels were produced, comprising six individuals and their technical replicates. The volume containing 0.5 μg of protein was diluted in IEF rehydration buffer [8 M Urea, 2 M Thiourea, 4% CHAPS, 0.0025% bromophenol blue, 65 mM DTT and 1% BioLyte 3-10 buffer 100 × (Bio-Rad, Hercules, CA)] to a final volume of 125 µl. After homogenization under continuous agitation for 1 hour at room temperature, the samples were centrifuged at 16,000 × g for 30 min. The supernatants were loaded onto 7 cm IPG strip 3-10NL pH range (Bio-Rad) by in-gel sample rehydration. Isoelectric focusing was carried out in a Protean IEF Cell (Bio-Rad) at 20°C and 50 µA/strip. Passive rehydration was performed for 4 hours, followed by active rehydration at 50 V for 12 hours, and focalization at 500 V for 30 min, followed by 1,000 V for 30 min, 4,000 V for 1 hour and 4,000 V up to 16,000 V/h. The IPG strips were equilibrated in reducing buffer (6 M Urea, 30% glycerol, 2% SDS, 50 mM Tris-HCl pH 8.8, 0.001% bromophenol blue and 130 mM DTT) for 10 min, and in alkylating buffer containing 135 mM iodoacetamide for a further 10 min. The IPG strips and molecular weight standard were placed on top of 12% SDS-PAGE gels and sealed with 0.5% agarose. The second dimension electrophoretic protein separation was carried out using a Mini-Protean III (Bio-Rad) under 60 V constant voltage for 10 min, and then under 100 V until the dye front reached the bottom of the gel. The Broad Range Molecular Weight (Bio-Rad, Hercules CA) was used as molecular marker. After electrophoresis, gels were stained by silver nitrate.

The 2D-gels were imaged using a GS-800 densitometer (Bio-Rad). The PDQuest software version 8.0.1 (Bio-Rad) was used to display protein spots in the 2D-gels, to create protein maps, and to perform qualitative comparative analysis, i.e. comparing the protein maps of patients within the same group to identify the spots belonging to the union set, or to the intersection subset of the group. The union set is defined as the ensemble of all spots observed in at least one of the 12 gels of the six subjects in a given group, while the intersection subset includes only the spots present in at least 11 out of the 12 gels of the group. Spots in the intersection subsets were considered the most representative of the respective groups.

### Protein identification by mass spectrometry

In order to obtain enough protein for mass spectrometry, 2D gels were prepared with pooled CSF samples of three patients with pneumococcal, meningococcal, or enteroviral meningitis, randomly selected from those used in the 2D PAGE described above. For each group of patients, three 2D gels were made containing 10, 40 and 100 μg protein, respectively, under the same conditions described above, but stained with Coomassie blue. Protein spots from the 2D PAGE were manually excised from the Coomassie blue stained gels and subjected to in-gel tryptic digestion. The resulting digest was analysed by matrix-assisted laser desorption/ionization-time of flight (MALDI-ToF-ToF) mass spectrometry using the analyser 5800 Proteomics (AB Sciex, Framingham, MA). The peptides were co-crystallized with 0.3 μl matrix of α-cyano-4-hydroxycinnamic acid (Sigma-Aldrich Inc., Saint Louis, MO) in 0.1% trifluoroacetic acid, and 50% acetonitrile directly on the MALDI plate. The MS and MS/MS data analysis were collected using a laser with an iteration rate of 1 kHz. The number of shots used was 2,000 for MS and MS/MS modes. The twelve most intense peaks whose signal-to-noise ratio was greater than two were selected as precursors to acquire MS/MS, except the peaks arising from trypsin autolysis or from the matrix. The MS external calibration mode was performed using a mixture of molecular mass standards containing des-arg1-bradykinin (m/z = 904.47), angiotensin I (m/z = 1296.69), Glu1-fibrinopeptide B (m/z = 1570.68) and adrenocorticotropic hormone (ACTH) (m/z = 3657.93) peptides. MS/MS spectra were externally calibrated using the known masses of the fragments observed in the MS/MS ion spectrum of Glu1-fibrinopeptide B. The list of peptide and fragment mass values generated by the mass spectrometer for each spot were submitted to a MS/MS ion search using MASCOT (Matrix Science, Boston, MA) to search in the *Homo sapiens *section of the NCBI Reference Sequence Database (NCBInr). The parameters used were: allowance of two tryptic miss cleavages, peptide error tolerance of ±0.6 Da, MS/MS error tolerance of ±0.2 Da, peptide charge +1 and variable modifications of methionine (oxidation), cysteine (carbamidomethylation and propionamidation). To avoid random matches, only ions with individual score above of the indicated by the MASCOT to identity or extensive homology (p < 0.05) were considered for protein identification.

### Bioinformatics analysis

The web version of the software SherLoc 2 (http://abi.inf.uni-tuebingen.de/Services/SherLoc2) was used to predict the subcellular localization of the proteins identified by mass spectrometry. SherLoc 2 integrates several types of sequence-derived and text-based information, and is suitable to a wide variety of eukaryotic organisms and subcellular localizations [49].

Pathway analysis was performed with the aid of the Enrichment Analysis (EA) workflow of the platform MetaCore of Thompson Reuters. For the purpose of the present study, the EA workflow can be defined as the attempt to match Swissprot protein IDs of the union sets of the groups with pneumococcal, meningococcal, or enteroviral meningitis with protein IDs in functional ontologies in MetaCore. The probability of a random intersection between a set of IDs the size of the user's list with ontology entities is estimated in P value of hypergeometric intersection. The lower *P *value means higher relevance of the entity to the dataset. In this study values smaller that 10^-5 ^were considered significant. Ontologies available for EA workflow include the Canonical Pathway maps (signalling and metabolic maps created by Thompson Reuters' scientists by manual curation of literature), Process Networks (cellular and molecular processes defined and annotated by Thompson Reuters scientists).

## Competing interests

The authors declare that they have no competing interests.

## Authors' contributions

APC - has made substantial contributions to the acquisition and analysis of data, and has been involved in drafting the manuscript.

RASP, AC, and JP - has made substantial contributions to the acquisition and analysis of 2D-PAGE data.

AC, JP - have made substantial contributions to the acquisition and analysis of MS data.

CSC - has made a substantial contribution to the setting up of the 2D-PAGE method for CSF and has participated in MS data acquisition.

GO - has contributed to the conception of the study.

TMSC - collected clinical data and CSF samples.

RSV - conceived, designed and coordinated this study, has made substantial contributions to acquisition, analysis and interpretation of data, and wrote the manuscript.

## Supplementary Material

Additional file 1 **2D gels of the six patients with pneumococcal meningitis**. Figure showing the images of one 2D PAGE gel of each patient (panels A to F) from the group of pneumococcal meningitis. MW = Molecular weight (Broad-range - BioRad). Click here for file

Additional file 2 **2D gels of the six patients with meningococcal meningitis**. Figure showing the images of one 2D PAGE gel of each patient (panels A to F) from the group of meningococcal meningitis. MW = Molecular weight (Broad-range - BioRad). Click here for file

Additional file 3 **2D gels of the six patients with enteroviral meningitis**. Figure showing the images of one 2D PAGE gel of each patient (panels A to F) from the group of enteroviral meningitis. MW = Molecular weight (Broad-range - BioRad). Click here for file

Additional file 4 **2D gels of the six control individuals**. Figure showing the images of one 2D PAGE gel of each patient (panels A to F) from the group of control individuals. MW = Molecular weight (Broad-range - BioRad). Click here for file

Additional file 5 **Additional Table 1 - Distribution of the spots and respective proteins of the intersection subset of patients with pneumococcal meningitis**. This table shows the distribution of the spots and respective proteins of the intersection subset of patients with pneumoccal meningitis Click here for file

Additional file 6 **Additional Table 2 - Distribution of the spots and respective proteins of the intersection subset of patients with menigococcal meningitis**. This table shows the distribution of the spots and respective proteins of the intersection subset of patients with menigococcal meningitis Click here for file

Additional file 7 **Additional Table 3 - Distribution of the spots and respective proteins of the intersection subset of patients with enteroviral meningitis**. This table shows the distribution of the spots and respective proteins of the intersection subset of patients with enteroviral meningitis Click here for file

Additional file 8 **Additional Table 4 - List of all protein identified by mass spectrometry**. This table contains the identification and description of all proteins identified by mass spectrometry, as well as their distribution in the union sets of the groups of patients with meningococcal, pneumococcal or enteroviral meningitis, and the control group. Click here for file

## Declarations

Publication costs for this article were funded by FIOCRUZ.

This article has been published as part of *BMC Genomics *Volume 16 Supplement 5, 2015: Proceedings of the 10th International Conference of the Brazilian Association for Bioinformatics and Computational Biology (X-Meeting 2014). The full contents of the supplement are available online at http://www.biomedcentral.com/bmcgenomics/supplements/16/S5.
