# Supplementary figures and images for: Comparative proteomics of cerebrospinal fluid reveals a predictive model for differential diagnosis of pneumococcal, meningococcal, and enteroviral meningitis, and novel putative therapeutic targets
Source: BMC Genomics. 2015 May 26;16(Suppl 5):S11. doi: 10.1186/1471-2164-16-S5-S11 (PMC4460676; doi:10.1186/1471-2164-16-S5-S11)

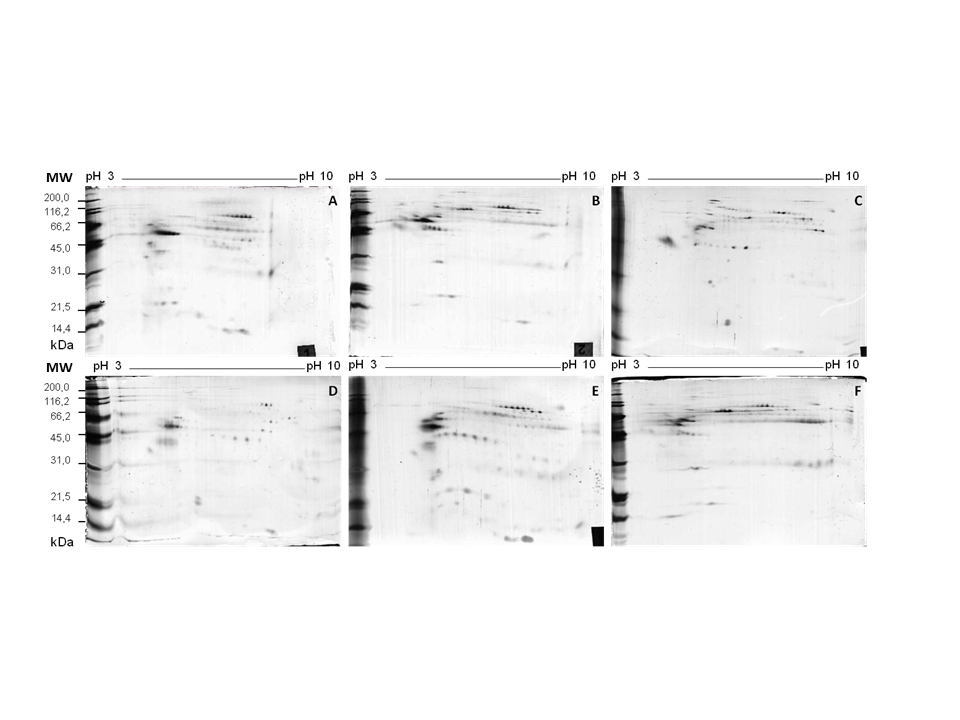

Supplement: Additional file 1 — 2D gels of the six patients with pneumococcal meningitis. Figure showing the images of one 2D PAGE gel of each patient (panels A to F) from the group of pneumococcal meningitis. MW = Molecular weight (Broad-range - BioRad). [file 1471-2164-16-S5-S11-S1.tif]

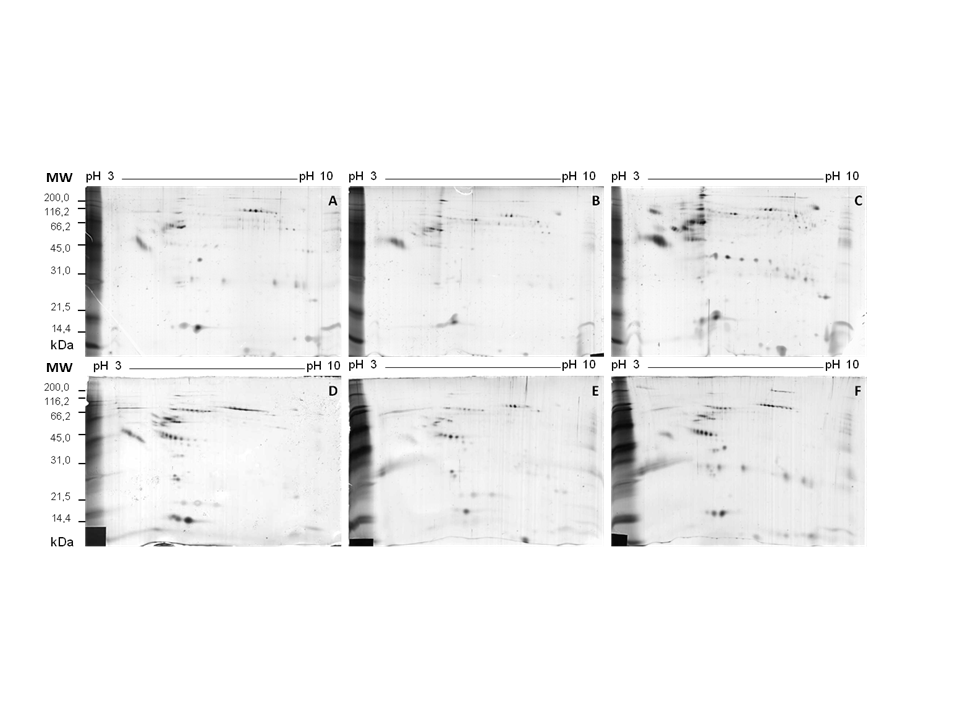

Supplement: Additional file 2 — 2D gels of the six patients with meningococcal meningitis. Figure showing the images of one 2D PAGE gel of each patient (panels A to F) from the group of meningococcal meningitis. MW = Molecular weight (Broad-range - BioRad). [file 1471-2164-16-S5-S11-S2.tif]

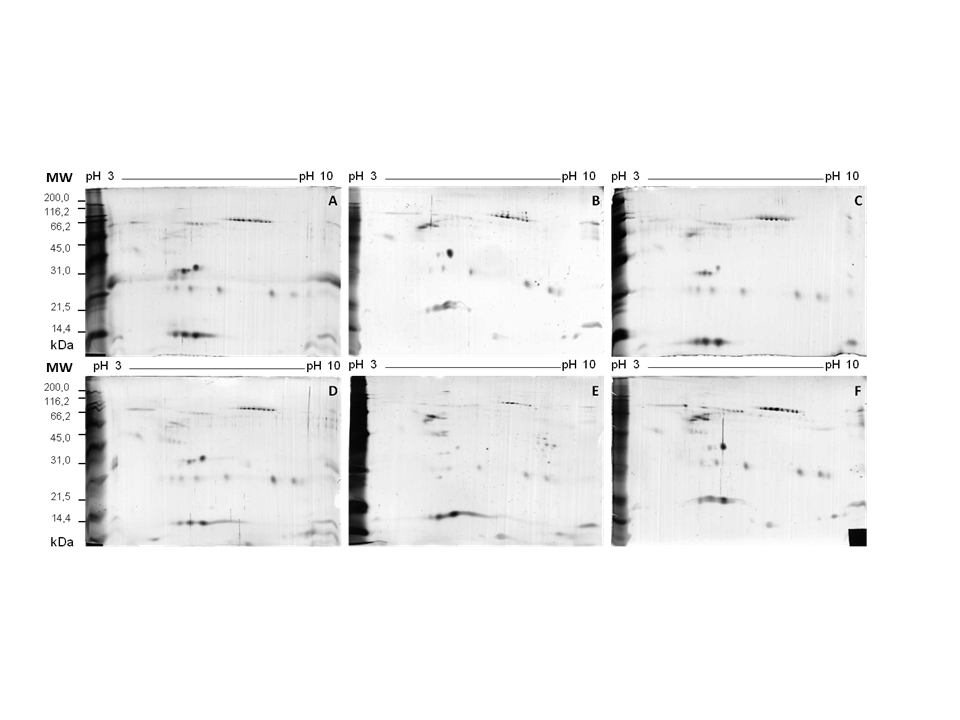

Supplement: Additional file 3 — 2D gels of the six patients with enteroviral meningitis. Figure showing the images of one 2D PAGE gel of each patient (panels A to F) from the group of enteroviral meningitis. MW = Molecular weight (Broad-range - BioRad). [file 1471-2164-16-S5-S11-S3.tif]

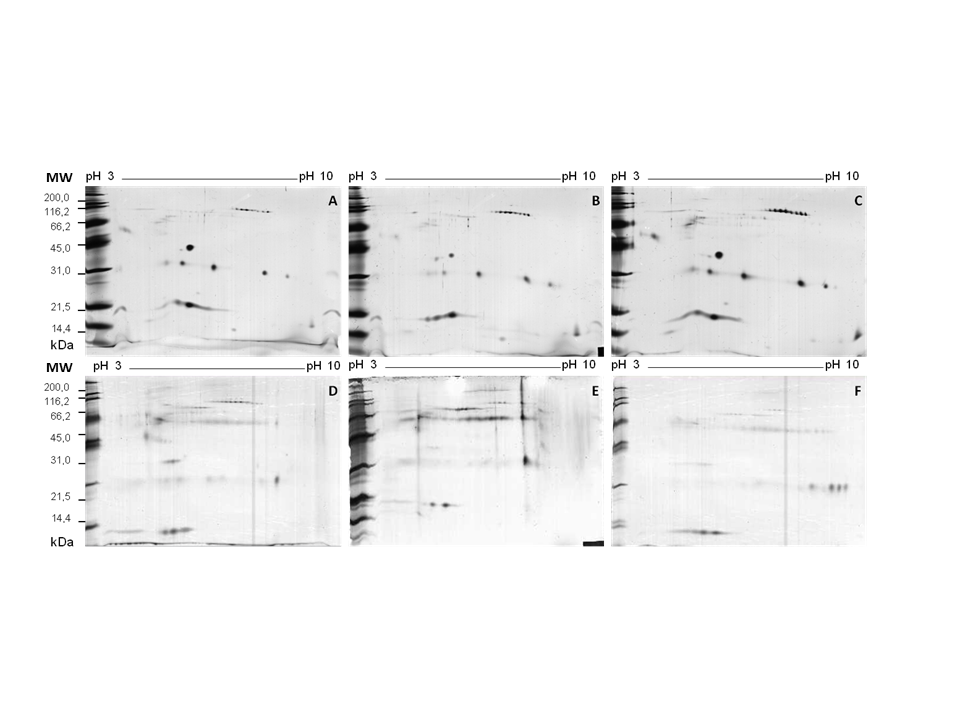

Supplement: Additional file 4 — 2D gels of the six control individuals. Figure showing the images of one 2D PAGE gel of each patient (panels A to F) from the group of control individuals. MW = Molecular weight (Broad-range - BioRad). [file 1471-2164-16-S5-S11-S4.tif]
