# Supplementary material for: Comparative proteomics of cerebrospinal fluid reveals a predictive model for differential diagnosis of pneumococcal, meningococcal, and enteroviral meningitis, and novel putative therapeutic targets
Source: BMC Genomics. 2015 May 26;16(Suppl 5):S11. doi: 10.1186/1471-2164-16-S5-S11 (PMC4460676; doi:10.1186/1471-2164-16-S5-S11)
Supplement: Additional file 5 — Additional Table 1 - Distribution of the spots and respective proteins of the intersection subset of patients with pneumococcal meningitis. This table shows the distribution of the spots and respective proteins of the intersection subset of patients with pneumoccal meningitis [file 1471-2164-16-S5-S11-S5.docx]

| Spots | | | | | | |  | Proteins |  | | | | |
| --- | --- | --- | --- | --- | --- | --- | --- | --- | --- | --- | --- | --- | --- |
| Spot | Definition | ∩ MM | ∩ MV | U MM | U MV | U Ctrl |  | NCBI (gi) | ∩ MM | ∩ MV | U MM | U MV | U Ctrl |
| p.1 | Alpha-2-HS-glycoprotein | 0 | 0 | 0 | 0 | 1 |  | 112910 | 0 | 0 | 1 | 1 | 1 |
| p.2 | Alpha-1-Antitrypsin | 0 | 0 | 0 | 0 | 1 |  | 6137432 | 1 | 1 | 1 | 1 | 1 |
| p.3 | Alpha-1-Antitrypsin | 0 | 0 | 1 | 1 | 1 |  | 6137432 | 1 | 1 | 1 | 1 | 1 |
| p.4 | Alpha-1-Antitrypsin | 0 | 0 | 1 | 1 | 1 |  | 6137432 | 1 | 1 | 1 | 1 | 1 |
| p.5 | Alpha-1-Antitrypsin | 0 | 0 | 0 | 0 | 0 |  | 6137432 | 1 | 1 | 1 | 1 | 1 |
| p.7 | Alpha-1-Antitrypsin | 0 | 0 | 1 | 1 | 1 |  | 6137432 | 1 | 1 | 1 | 1 | 1 |
| p.6 | protein Tro alpha1 H | 1 | 0 | 1 | 1 | 1 |  | 223069 | 1 | 0 | 1 | 0 | 1 |
| p.8 | Hemopexin precursor | 0 | 0 | 1 | 1 | 1 |  | 386789 | 1 | 1 | 1 | 1 | 1 |
| p.9 | Hemopexin precursor | 0 | 0 | 1 | 1 | 1 |  | 386789 | 1 | 1 | 1 | 1 | 1 |
| p.10 | Hemopexin precursor | 0 | 0 | 1 | 1 | 1 |  | 386789 | 1 | 1 | 1 | 1 | 1 |
| p.11 | Serum albumin | 0 | 0 | 1 | 1 | 1 |  | 28592 | 1 | 0 | 1 | 1 | 1 |
| p.12 | Non identified | 0 | 0 | 1 | 1 | 1 |  | - | 0 | 0 | 1 | 1 | 1 |
| p.13 | Transferrin | 0 | 0 | 1 | 1 | 1 |  | 115394517 | 1 | 1 | 1 | 1 | 1 |
| p.14 | Transferrin | 0 | 0 | 1 | 1 | 1 |  | 115394517 | 1 | 1 | 1 | 1 | 1 |
| p.15 | Transferrin | 0 | 0 | 1 | 1 | 1 |  | 115394517 | 1 | 1 | 1 | 1 | 1 |
| p.16 | Complement C3 | 1 | 0 | 1 | 0 | 1 |  | 179665 | 1 | 0 | 1 | 0 | 1 |
| p.17 | Haptoglobin precursor | 0 | 0 | 1 | 1 | 1 |  | 306882 | 1 | 0 | 1 | 1 | 1 |
| p.18 | Haptoglobin precursor | 0 | 0 | 1 | 1 | 1 |  | 306882 | 1 | 0 | 1 | 1 | 1 |
| p.19 | Haptoglobin precursor | 0 | 0 | 1 | 1 | 1 |  | 306882 | 1 | 0 | 1 | 1 | 1 |
| p.20 | C-reactive protein | 1 | 0 | 1 | 0 | 1 |  | 1942435 | 1 | 0 | 1 | 0 | 1 |
| p.21 | Apolipoprotein A-I | 1 | 1 | 1 | 1 | 0 |  | 90108664 | 1 | 1 | 1 | 1 | 0 |
| p.22 | Transthyretin | 1 | 0 | 1 | 0 | 1 |  | 17942890 | 1 | 1 | 1 | 1 | 1 |
| p.23 | Transthyretin | 0 | 0 | 0 | 0 | 0 |  | 17942890 | 1 | 1 | 1 | 1 | 1 |

∩ = intersection subset; U = union set; MM = menigococcal meningitis; MV = enteroviral meningitis; Ctrl: control; 1 = present; 0 = absent.
