# Supplementary material for: Comparative proteomics of cerebrospinal fluid reveals a predictive model for differential diagnosis of pneumococcal, meningococcal, and enteroviral meningitis, and novel putative therapeutic targets
Source: BMC Genomics. 2015 May 26;16(Suppl 5):S11. doi: 10.1186/1471-2164-16-S5-S11 (PMC4460676; doi:10.1186/1471-2164-16-S5-S11)
Supplement: Additional file 6 — Additional Table 2 - Distribution of the spots and respective proteins of the intersection subset of patients with menigococcal meningitis. This table shows the distribution of the spots and respective proteins of the intersection subset of patients with menigococcal meningitis [file 1471-2164-16-S5-S11-S6.docx]

| Spots | | | | | | |  | Proteins |  |  | | | |
| --- | --- | --- | --- | --- | --- | --- | --- | --- | --- | --- | --- | --- | --- |
| Spot | Definition | ∩ MP | ∩ MV | U MP | U MV | U Ctrl |  | NCBI (gi) | ∩ MP | ∩ MV | U MP | U MV | U Ctrl |
| m.1 | Ceruloplasmin | 0 | 0 | 1 | 0 | 1 |  | 1620909 | 0 | 0 | 0 | 1 | 1 |
| m.2 | Alpha-1-B-glycoprotein | 0 | 0 | 1 | 0 | 0 |  | 69990 | 0 | 0 | 1 | 1 | 1 |
| m.3 | Alpha-1-B-glycoprotein | 0 | 0 | 1 | 1 | 1 |  | 69990 | 0 | 0 | 1 | 1 | 1 |
| m.4 | T Cell Receptor Beta Chains Related  To Rheumatoid Arthritis | 0 | 0 | 1 | 0 | 0 |  | 78101492 | 1 | 1 | 1 | 1 | 0 |
| m.9 | T Cell Receptor Beta Chains Related  To Rheumatoid Arthritis | 1 | 1 | 1 | 1 | 0 |  | 78101492 | 1 | 1 | 1 | 1 | 0 |
| m.5 | Hemopexin precursor | 0 | 0 | 1 | 0 | 1 |  | 386789 | 1 | 1 | 1 | 1 | 1 |
| m.6 | Hemopexin precursor | 0 | 0 | 1 | 1 | 1 |  | 386789 | 1 | 1 | 1 | 1 | 1 |
| m.7 | Hemopexin precursor | 0 | 0 | 0 | 1 | 1 |  | 386789 | 1 | 1 | 1 | 1 | 1 |
| m.8 | Hemopexin precursor | 0 | 0 | 1 | 1 | 1 |  | 386789 | 1 | 1 | 1 | 1 | 1 |
| m.10 | Transferrin | 1 | 1 | 1 | 1 | 1 |  | 115394517 | 1 | 1 | 1 | 1 | 1 |
| m.11 | Transferrin | 1 | 1 | 1 | 1 | 0 |  | 115394517 | 1 | 1 | 1 | 1 | 1 |
| m.12 | Transferrin | 1 | 1 | 1 | 1 | 1 |  | 115394517 | 1 | 1 | 1 | 1 | 1 |
| m.13 | Transferrin | 1 | 1 | 1 | 1 | 1 |  | 115394517 | 1 | 1 | 1 | 1 | 1 |
| m.14 | Transferrin | 1 | 0 | 1 | 0 | 0 |  | 115394517 | 1 | 1 | 1 | 1 | 1 |
| m.15 | Alpha-1-antichymotrypsin precursor | 1 | 0 | 1 | 0 | 1 |  | 177933 | 1 | 0 | 1 | 0 | 1 |
| m.16 | Alpha-1-Antitrypsin | 1 | 1 | 1 | 1 | 0 |  | 6137432 | 1 | 1 | 1 | 1 | 1 |
| m.17 | Kininogen-1 | 0 | 0 | 0 | 0 | 0 |  | 4504893 | 0 | 0 | 0 | 0 | 0 |
| m.18 | serum vitamin D-binding protein precursor | 1 | 1 | 1 | 1 | 0 |  | 181482 | 1 | 1 | 1 | 1 | 1 |
| m.19 | serum vitamin D-binding protein precursor | 1 | 0 | 1 | 0 | 0 |  | 181482 | 1 | 1 | 1 | 1 | 1 |
| m.20 | Alpha-1-Antitrypsin | 0 | 0 | 0 | 0 | 0 |  | 6137432 | 1 | 1 | 1 | 1 | 1 |
| m.21 | Alpha-1-Antitrypsin | 1 | 1 | 1 | 1 | 0 |  | 6137432 | 1 | 1 | 1 | 1 | 1 |
| m.22 | Alpha-1-acid glycoprotein | 0 | 0 | 0 | 0 | 0 |  | 112877 | 1 | 1 | 1 | 1 | 1 |
| m.23 | Alpha-1-acid glycoprotein | 1 | 0 | 1 | 0 | 0 |  | 112877 | 1 | 1 | 1 | 1 | 1 |
| m.24 | Haptoglobin precursor | 0 | 0 | 0 | 1 | 0 |  | 306882 | 1 | 0 | 1 | 1 | 1 |
| m.27 | Haptoglobin precursor | 1 | 0 | 1 | 1 | 0 |  | 306882 | 1 | 0 | 1 | 1 | 1 |
| m.28 | Haptoglobin precursor | 1 | 0 | 1 | 1 | 1 |  | 306882 | 1 | 0 | 1 | 1 | 1 |
| m.25 | Complement C3 | 0 | 0 | 0 | 0 | 0 |  | 179665 | 1 | 0 | 1 | 0 | 1 |
| m.26 | Zn-alpha2-glycoprotein | 0 | 0 | 1 | 1 | 1 |  | 38026 | 0 | 0 | 1 | 1 | 1 |
| m.29 | C-reactive protein | 0 | 0 | 1 | 0 | 0 |  | 1942435 | 0 | 0 | 1 | 0 | 1 |
| m.30 | Apolipoprotein A-I | 0 | 0 | 0 | 0 | 0 |  | 90108664 | 1 | 1 | 1 | 1 | 0 |
| m.31 | Apolipoprotein A-I | 1 | 1 | 1 | 1 | 0 |  | 90108664 | 1 | 1 | 1 | 1 | 0 |
| m.32 | Transthyretin | 0 | 1 | 0 | 1 | 0 |  | 17942890 | 1 | 1 | 1 | 1 | 1 |
| m.33 | Transthyretin | 0 | 1 | 0 | 1 | 0 |  | 17942890 | 1 | 1 | 1 | 1 | 1 |

∩ = intersection subset; U = union set; MP = pneumococcal meningitis; MV = enteroviral meningitis; Ctrl: control; 1 = present; 0 = absent.
