# Supplementary material for: Comparative proteomics of cerebrospinal fluid reveals a predictive model for differential diagnosis of pneumococcal, meningococcal, and enteroviral meningitis, and novel putative therapeutic targets
Source: BMC Genomics. 2015 May 26;16(Suppl 5):S11. doi: 10.1186/1471-2164-16-S5-S11 (PMC4460676; doi:10.1186/1471-2164-16-S5-S11)
Supplement: Additional file 7 — Additional Table 3 - Distribution of the spots and respective proteins of the intersection subset of patients with enteroviral meningitis. This table shows the distribution of the spots and respective proteins of the intersection subset of patients with enteroviral meningitis [file 1471-2164-16-S5-S11-S7.docx]

| Spots | | | | | | |  | Proteins |  |  | | | |
| --- | --- | --- | --- | --- | --- | --- | --- | --- | --- | --- | --- | --- | --- |
| Spot | Definition | ∩ MP | ∩ MM | U MP | U MM | U Ctrl |  | NCBI (gi) | ∩ MP | ∩ MM | U MP | U MM | U Ctrl |
| v.1 | Non identified | 0 | 0 | 0 | 0 | 0 |  | - | 0 | 0 | 0 | 0 | 0 |
| v.2 | Non identified | 0 | 0 | 0 | 1 | 0 |  | - | 0 | 0 | 0 | 1 | 0 |
| v.3 | Non identified | 0 | 0 | 0 | 0 | 0 |  | - | 0 | 0 | 0 | 0 | 0 |
| v.4 | Non identified | 0 | 0 | 1 | 1 | 1 |  | - | 0 | 0 | 1 | 1 | 1 |
| v.5 | unnamed protein product | 0 | 0 | 1 | 1 | 1 |  | 22761380 | 0 | 0 | 1 | 1 | 1 |
| v.6 | Hemopexin precursor | 0 | 0 | 1 | 1 | 1 |  | 386789 | 1 | 1 | 1 | 1 | 1 |
| v.7 | Non identified | 0 | 0 | 1 | 1 | 1 |  | - | 0 | 0 | 1 | 1 | 1 |
| v.8 | Hemopexin precursor | 0 | 0 | 1 | 1 | 0 |  | 386789 | 1 | 1 | 1 | 1 | 1 |
| v.9 | unnamed protein product | 0 | 0 | 1 | 1 | 1 |  | 22761380 | 0 | 0 | 1 | 1 | 1 |
| v.10 | Transferrin | 0 | 0 | 0 | 1 | 1 |  | 110590597 | 1 | 1 | 1 | 1 | 1 |
| v.11 | Transferrin | 0 | 0 | 1 | 1 | 1 |  | 110590597 | 1 | 1 | 1 | 1 | 1 |
| v.12 | Transferrin | 0 | 0 | 1 | 1 | 1 |  | 110590597 | 1 | 1 | 1 | 1 | 1 |
| v.13 | Transferrin | 0 | 0 | 1 | 1 | 1 |  | 110590597 | 1 | 1 | 1 | 1 | 1 |
| v.14 | Non identified | 0 | 0 | 1 | 1 | 1 |  | - | 0 | 0 | 1 | 1 | 1 |
| v.15 | Transferrin | 0 | 0 | 1 | 1 | 1 |  | 110590597 | 1 | 1 | 1 | 1 | 1 |
| v.16 | Transferrin | 0 | 0 | 1 | 0 | 1 |  | 110590597 | 1 | 1 | 1 | 1 | 1 |
| v.17 | Alpha-1-Antitrypsin | 0 | 0 | 0 | 1 | 1 |  | 177831 | 1 | 1 | 1 | 1 | 1 |
| v.18 | Non identified | 0 | 0 | 1 | 0 | 1 |  | - | 0 | 0 | 0 | 0 | 1 |
| v.19 | serum vitamin D-binding protein precursor | 0 | 0 | 1 | 0 | 1 |  | 181482 | 1 | 1 | 1 | 1 | 1 |
| v.20 | serum vitamin D-binding protein precursor | 0 | 0 | 1 | 0 | 1 |  | 181482 | 1 | 1 | 1 | 1 | 1 |
| v.21 | Alpha-1-acid glycoprotein | 0 | 0 | 0 | 0 | 1 |  | 112877 | 1 | 1 | 1 | 1 | 1 |
| v.22 | Transthyretin | 0 | 0 | 1 | 0 | 1 |  | 17942890 | 1 | 1 | 1 | 1 | 1 |
| v.23 | Transthyretin | 0 | 0 | 0 | 1 | 1 |  | 17942890 | 1 | 1 | 1 | 1 | 1 |
| v.24 | Prostaglandin D synthase | 0 | 0 | 0 | 0 | 1 |  | 283806778 | 0 | 0 | 0 | 1 | 1 |
| v.25 | Prostaglandin D synthase | 0 | 0 | 0 | 0 | 0 |  | 283806778 | 0 | 0 | 0 | 1 | 1 |
| v.26 | Non identified | 0 | 0 | 0 | 1 | 0 |  | - | 0 | 0 | 0 | 1 | 0 |
| v.27 | Transthyretin | 0 | 0 | 0 | 0 | 0 |  | 17942890 | 1 | 1 | 1 | 1 | 1 |
| v.28 | Apolipoprotein A-I | 1 | 1 | 1 | 1 | 0 |  | 90108664 | 1 | 1 | 1 | 1 | 0 |

∩ = intersection subset; U = union set; MP = pneumococcal meningitis; MV = meningococal meningitis; Ctrl: control; 1 = present; 0 = absent.
